# A new suspect: *Listeria monocytogenes* outbreak linked to pasteurised plant-based beverages, Canada, 2024

**DOI:** 10.2807/1560-7917.ES.2026.31.24.2500883

**Published:** 2026-06-18

**Authors:** J Leigh Hobbs, Allana Murphy, Jackson Chung, Franco Pagotto, Meghan Hamel, Ashley Kearney, Natalie Fava, Rajesh Benny, Tharany Nadarajah, Shovita Padhi, Angela Catford, Antoine Corbeil, Anna Majury, Christine Navarro

**Affiliations:** 1 Public Health Ontario, Toronto, Canada; 2 Health Canada, Ottawa, Canada; 3 Public Health Agency of Canada, Ottawa, Canada; 4 Public Health Agency of Canada, Winnipeg, Canada; 5 Toronto Public Health, Toronto, Canada; 6 University of Toronto, Toronto, Canada; 7 Queen’s University, Kingston, Canada

**Keywords:** *Listeria monocytogenes*, Listeriosis, Outbreak, Foodborne illness, Plant-based beverages, Plant-based products, Dairy alternative products, Whole genome sequencing

## Abstract

In Canada in 2024, an outbreak of *Listeria monocytogenes* occurred, linked to pasteurised plant-based dairy alternative beverage products. The outbreak involved 20 confirmed cases (age range: 7–89 years; 13 females, seven males; 14 having underlying medical conditions) and resulted in three deaths. All cases were infected with the same genetic strain of *L. monocytogenes*. Nineteen of the 20 outbreak-confirmed cases reported consuming the same brand of plant-based beverage product prior to illness onset, and the outbreak strain was found in six open leftover plant-based beverage samples collected from the homes of six outbreak-confirmed cases. All *L. monocytogenes*-positive food samples were produced at a single manufacturing facility in Ontario, Canada on the same production line. The outbreak strain was confirmed in post-pasteurisation processing areas at the manufacturing facility. However, the source of contamination was not determined. Leftover case–food sample collection by local public health authorities and laboratory testing were instrumental in the timely identification of the outbreak source - a food recall warning was issued within 2 weeks of initial case–food WGS match. Manufacturer adherence to applicable requirements regarding *L. monocytogenes* in ready-to-eat foods continues to be critical to prevent *Listeria* contamination and subsequent illnesses and/or deaths.

**Figure:**
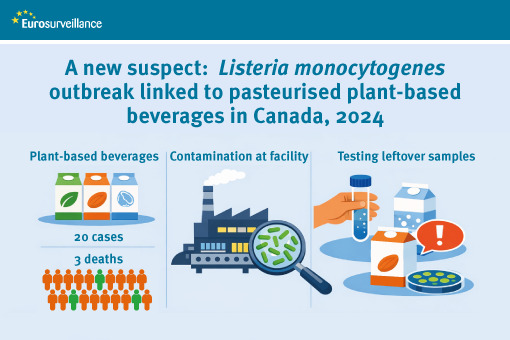

**Key public health message**

**What did you want to address in this study and why?**

Invasive listeriosis, caused by *Listeria monocytogenes* bacteria, is a rare but severe and potentially fatal illness typically occurring among individuals with weakened immune systems. We describe the evidence gathered to implicate a novel listeriosis outbreak source in an investigation that occurred in Ontario, Canada in 2024, as well as the measures implemented to prevent further illness related to this outbreak.

**What have we learnt from this study?**

The outbreak involved 20 laboratory-confirmed cases, and three deaths. All cases were infected with the same genetic strain of *L. monocytogenes*. Plant-based dairy alternative beverage products were implicated as the source of the outbreak, emphasising the importance of considering new sources that have not been previously implicated in *L. monocytogenes* outbreak investigations.

**What are the implications of your findings for public health?**

Leftover food sample collection and testing were instrumental in the timely identification of a novel outbreak source. While the source of contamination was not identified, manufacturer adherence to applicable regulations on *L. monocytogenes* in ready-to-eat foods, including plant-based beverage products, continues to be critical to prevent contamination and subsequent illnesses and/or deaths.

## Background

In Ontario, Canada, an average of 70 cases of invasive listeriosis, caused by infection with the pathogenic bacteria *Listeria monocytogenes*, are reported annually [1]. Invasive disease typically occurs among individuals with a weakened immune system, which may include those 70 years of age and older and pregnant people. Although rare, illness can often be severe or potentially fatal. Hospitalisation is reported for an average of 65% of cases annually and a fatal outcome is reported for 12% of cases, making listeriosis a leading cause of death from a food-borne illness in Ontario [1].

Although *L. monocytogenes* is ubiquitous in the environment, infection typically results from food-borne transmission. Introduction of *Listeria* in food processing environments can lead to contamination and the formation of biofilms can make the bacteria difficult to eliminate [2]. Ready-to-eat meats and soft cheeses are widely recognised sources of *L. monocytogenes* [3-6]. Novel sources continue to be identified, including whole uncut fresh produce, frozen produce and pasteurised products [7-13]. Plant-based products are not a well-known source of *L. monocytogenes*. An outbreak in 2022 in France was linked to plant-based cheeses, and another in 2018 in Finland was linked to fava bean products [14,15]. To date, no known outbreaks of *L. monocytogenes* have been linked to plant-based dairy-alternative beverage products, although studies have demonstrated *L. monocytogenes* growth in these types of products [16,17].

## Outbreak detection

On 24 June 2024, Public Health Ontario (PHO) was notified of a *L. monocytogenes* case isolate that was related by whole genome multilocus sequencing typing (wgMLST) to a positive open leftover food sample collected from the case’s home as part of routine follow up carried out by a local public health authority. The sample was a refrigerated coconut beverage sold under Brand A and past the expiry date; the isolated *L. monocytogenes* was genetically related to the case’s clinical isolate. The *L. monocytogenes*-positive coconut beverage was one of 14 various food and beverage samples collected for testing by the local public health authority from the case’s home. Samples were collected (following consultation with PHO’s laboratory) regardless of whether the food item was prompted for on the national listeriosis standardised questionnaire. *Listeria* was not detected in any of the other 13 samples collected from the case’s home, including a sample of almond beverage sold under the same Brand A.

Public Health Ontario was also notified that the case–food match was genetically related to three historical isolates in the national database, including two cases from Ontario and one from Nova Scotia (another Canadian province) with illness onset dates ranging from August to December 2023. These cases had previously been investigated, however, with no clear hypothesis a source of illness was not identified. Further, three newly sequenced Ontario cases were genetically related to the cluster for a total of seven cases with onset dates spanning an eleven-month period from August 2023 to June 2024. As the situation appeared to be active only in Ontario, PHO led the investigation and an Ontario Outbreak Investigation Coordinating Committee (ON-OICC) was established to facilitate coordination across multiple agencies and investigate plant-based beverage products as a possible source of illness.

This report describes the investigation that was conducted in Ontario, Canada that linked an outbreak of 20 genetically related *L. monocytogenes* cases to pasteurised plant-based beverage products, and highlights evidence gathered to implicate plant-based beverage products as the source of illness, as well as outbreak control measures.

## Methods

### Routine case finding

In Ontario, Canada, invasive listeriosis is designated a disease of public health significance. Cases of illness are reported under provincial legislation to local public health authorities [18]. Local public health investigators routinely follow up with cases using a national standardised questionnaire to identify possible sources of disease acquisition in the 4 weeks before illness onset. Case details are recorded through the Ontario Ministry of Health (MOH)-integrated Public Health Information System (iPHIS), the reportable disease database for the province.

Local public health investigators may also collect leftover (i.e. open non-intact) samples of food items consumed by cases in the 4 weeks before illness onset as well as control (i.e. closed intact) samples from the same lot, and submit those samples for testing at PHO.

### Outbreak detection and management

Public Health Ontario carries out routine surveillance and monitoring of listeriosis at the provincial level for the purposes of cluster identification and outbreak detection. When a multi-jurisdictional outbreak within Ontario (i.e. involving cases from two or more local public health authorities) is detected, PHO leads the investigation and, when coordination across multiple agencies is required, establishes an ON-OICC [19]. When an outbreak within Canada involving cases from two or more provinces or territories is detected, the Public Health Agency of Canada (PHAC) leads the investigation.

Public Health Ontario may issue directions to local public health authorities to request enhanced case follow up, including administration of a focused questionnaire and targeted case–food sample collection. In this investigation, as the case–food match identified a possible source of illness, a plant-based dairy-alternative product-focused questionnaire was developed (Supplement S1). The focused questionnaire prompted for different dairy-alternative products (e.g. beverages, cheeses, sour creams) of various types (e.g. coconut, almond, oat, soy) consumed in the four weeks before illness onset. The questionnaire further prompted for product details (e.g. brand, flavour, production codes), photos of all sides of product packaging, and to collect any leftover products for testing.

### Outbreak case definition

An outbreak-confirmed case was defined as a resident or visitor to Canada with laboratory confirmation of *L. monocytogenes* with genetically related clinical isolates by WGS (i.e. within approximately 10 allelic differences by wgMLST), and symptom onset on or after 1 August 2023. Laboratory-confirmed cases of listeriosis are considered invasive as isolation of *L. monocytogenes* from a normally sterile site (e.g. blood) is required.

### Public Health Ontario’s laboratory testing methods

Community and hospital laboratories in Ontario submit *L. monocytogenes* clinical isolates to PHO’s laboratory for subtyping by wgMLST. Whole genome sequencing (WGS) analysis is performed using standardised PulseNet Canada pipelines and compared with both provincial databases (preliminary result) and national databases (confirmatory result) to identify clusters of genetically related isolates. Confirmation is completed by PHAC National Microbiology Laboratory Branch (NMLB).

Further, PHO’s laboratory analyses food samples collected by local public health investigators in relation to confirmed cases of listeriosis. Real-time PCR is used for the detection of *L. monocytogenes* in food [20]. Food samples where *L. monocytogenes* is detected by PCR are further analysed by routine culture [21]. Culture-positive isolates are subsequently sequenced by WGS in accordance with PulseNet Canada standard operating procedures [22].

### Environmental traceback and investigation

The Canadian Food Inspection Agency (CFIA) is notified of *Listeria*-positive food items collected in relation to a confirmed case. The CFIA leads the food safety investigation which involves tracing *Listeria*-positive products to the manufacturing facility, obtaining distribution information, reviewing records and processes, and conducting representative sampling of closed intact (e.g. retention) samples of the implicated food item.

Environmental swabbing may also be conducted to determine the source and scope of the contamination. Closed food samples and environmental swabs collected by the CFIA are analysed at CFIA’s laboratories according to Health Canada’s Compendium of Analytic methods. *L. monocytogenes*-positive isolates are sequenced by WGS in accordance with PulseNet Canada standard operating procedures. Risk mitigating action, such as a public food recall warning where an implicated product is withdrawn from the market, may be requested [23].

### Health risk assessment

Public Health Ontario may submit an assessment describing the evidence gathered in the epidemiological investigation implicating a specific product as the source of illness for an outbreak. As part of the Health Risk Assessment (HRA) process, the epidemiological assessment is reviewed by Health Canada (along with submissions describing evidence gathered in the food safety investigation) to determine if there is sufficient evidence to implicate the suspected product as the source of illness [24]. As an outcome of the HRA, the CFIA may issue a food recall warning.

### Health Canada’s laboratory testing methods

Health Canada’s laboratory can perform enumeration and bacterial growth studies for *L. monocytogenes* in food products. Closed food samples were analysed using Health Canada method MFLP-01 for the presence of *L. monocytogenes* [21]. Challenge studies using *L. monocytogenes* growth were done by artificially inoculating samples with either a high (1,000 colony-forming units or CFU) or low (100 CFU) amount of *L. monocytogenes* and storing them at 7 °C with colony counts at day 7 and day 68.

## Results

### Epidemiological investigation

Exposure information collected during routine follow-up using the listeriosis national standardised questionnaire was reviewed for the Ontario listeriosis outbreak-confirmed cases to determine a possible source of illness. Details were requested for the Nova Scotia case. No commonalities among reported exposure details were identified. However, the national standardised questionnaire did not include questions related to plant-based dairy-alternative products. Thus, aside from the case–food match, this information was not available for any of the other cases.

Public Health Ontario developed a listeriosis plant-based dairy-alternative product-focused questionnaire to collect this information for all cases. Local public health investigators were directed to administer the focused questionnaire to both *L. monocytogenes* cases that were genetically related to the outbreak as well as confirmed *L. monocytogenes* cases where WGS was pending (i.e. where it was not yet known if the case was related to the outbreak strain). Public Health Ontario requested that health authorities in Nova Scotia re-interview their case using the same focused questionnaire.

### Outbreak description

**Figure 1:**
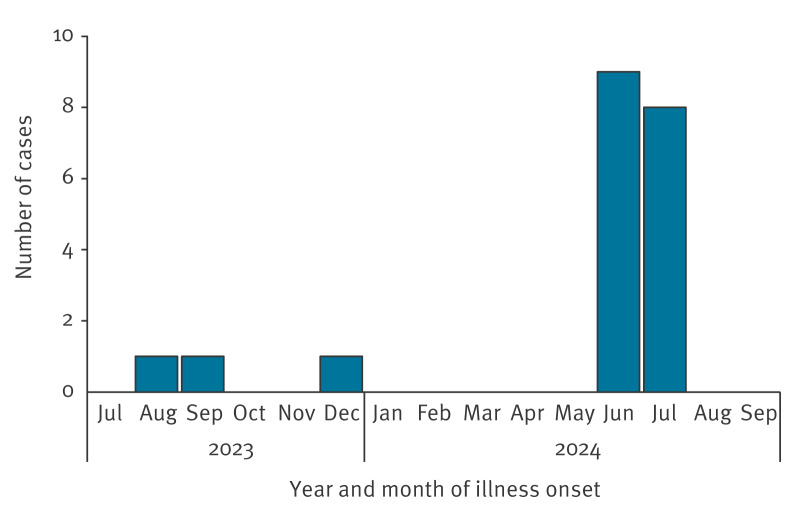
Outbreak-confirmed cases of *Listeria monocytogenes* by month of illness onset, Canada, July 2023–September 2024 (n = 20)

A total of 20 outbreak-confirmed cases from four Canadian provinces were identified, with most cases (n = 13 cases) reported in Ontario (Figure 1). Illness onset dates ranged from August 2023 to July 2024, with 17 cases occurring in June and July of 2024. Thirteen outbreak-confirmed cases were female and seven were male. Ages ranged from 7 to 89 years and 14 were 50 years or older. Fourteen cases reported an underlying medical condition. No outbreak-confirmed cases were pregnant. Fifteen hospitalisations and three deaths were reported.

The case–food match was detected in June 2024.

**Figure 2:**
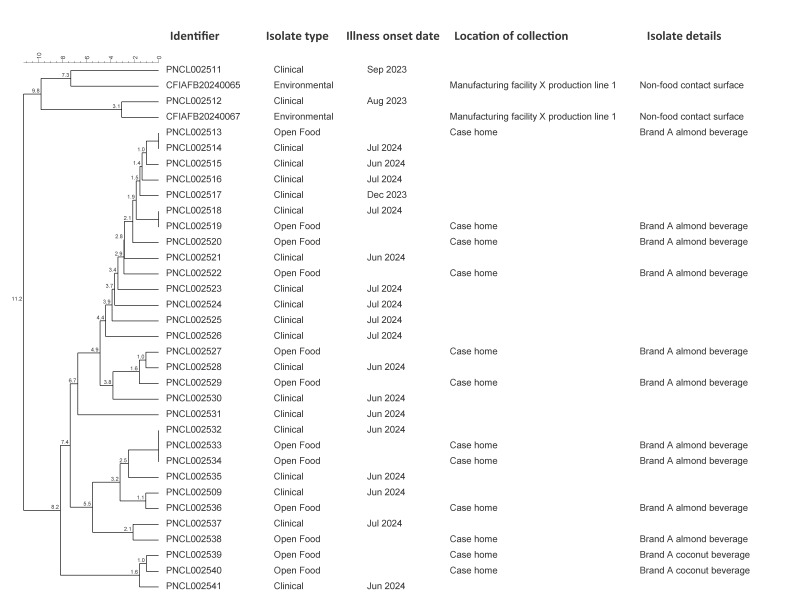
Whole genome sequence typing (wgMLST) analysis of clinical, food and environmental isolates from the *Listeria monocytogenes* outbreak linked to plant-based beverage products, Canada, 2023–2024 (n = 34)

Clinical isolates were genetically related and clustered within 11 wgMLST alleles (Figure 2). No international isolates related to the cluster were identified.

### Traceback and investigation at the manufacturing facility

Details of the *L. monocytogenes*-positive leftover coconut beverage sample were shared with the CFIA. The CFIA determined that Brand A used several different manufacturing facilities located in North America for their products. However, production codes indicated the *L. monocytogenes*-positive Brand A coconut beverage was produced at a manufacturing facility (Manufacturer X) located in Ontario using a dedicated closed-system production line (Line 1). The product was pasteurised (i.e. heated to high temperatures) with no additional ingredients added post-pasteurisation. Line 1 plant-based beverage products were distributed nationally, with the majority in Ontario.

### Focused case investigation

By 5 July 2024, nine Ontario outbreak-confirmed cases had been reported. Focused re-interviews were completed for eight of the nine Ontario cases. All eight re-interviewed Ontario cases (including the cases from 2023) reported consuming Brand A plant-based beverage products. No commonalities were reported for any other plant-based dairy-alternative products. The proportion of re-interviewed Ontario outbreak-confirmed cases reporting plant-based beverage products (100%) was statistically significant (p value < 0.05) when compared with Foodbook 2.0 estimates for consumption of non-dairy milk substitutes for the Ontario general population (21%) [25]. However, due to the small sample size, the statistical association should be interpreted with caution. *Listeria monocytogenes* was also detected by PCR in two leftover Brand A almond beverage samples collected from the homes of two Ontario outbreak-confirmed cases. Culture and WGS were pending for both samples. Both products were produced at Manufacturing Facility X on Line 1. *Listeria* was not detected by PCR in a closed retail sample of Brand A coconut beverage produced by Manufacturing Facility X on Line 1, nor was it detected in Brand A leftover case samples of dairy-alternative products produced by different manufacturing facilities. However, given the strength of the evidence, Manufacturing Facility X ceased production on Line 1. The existing product was detained and no further distribution occurred.

## Outbreak control measures

On 6 July 2024, PHO submitted an epidemiological assessment implicating Brand A plant-based beverages produced at Manufacturing Facility X on Line 1 as the source of illness for the outbreak, triggering the HRA process. However, the process was not completed as in the interim the company took action and initiated a voluntary public food recall warning posted on 8 July 2024 [26]. The recall was large in scope and included 18 different Brand A refrigerated beverage product types and flavours with production dates up to and including October 2024. A second brand (Brand B) of plant-based refrigerated beverages that was produced at the same manufacturing facility on the same line was also included in the recall warning. No outbreak-confirmed cases reported consuming Brand B products produced at Manufacturing Facility X. However, given the potential for contamination, Brand B products were included in the recall warning. The CFIA carried out recall effectiveness checks, confirming recalled products were removed from the market. On 9 July 2024, Ontario’s Chief Medical Officer of Health issued a public statement warning Ontarians not to consume the recalled products due to *Listeria* contamination [27].

### Post-recall findings

Following the recall warning, an additional 10 outbreak-confirmed cases were identified, including four cases from Ontario, five cases from Quebec and one case from Alberta, for a total of 20 listeriosis outbreak-confirmed cases across Canada. These newly identified cases met the outbreak case definition and were re-interviewed. Overall, 19 of the 20 cases reported consuming Brand A plant-based beverage products, further confirming Brand A plant-based beverages as the source of illness and strengthening evidence in support of the existing recall. The PHAC issued public communications at the national level warning Canadians not to consume the recalled products [28]. Further, PHO’s laboratory completed food testing for additional Brand A leftover case samples, as well as Brand A closed retail samples. A total of six open case samples of Brand A plant-based beverage products produced by Manufacturing Facility X on Line 1 were confirmed to be a genetic match to the cluster (Table). The samples included almond and coconut beverages of various flavours with best before dates ranging from early July to mid-September 2024. Three closed Brand A plant-based beverage product retail samples produced on Line 1 were also tested; *Listeria* was not detected in any of the closed retail samples.

**Table:** Samples of Brand A plant-based beverage products produced by Manufacturing Facility X on Line 1 tested at the Public Health Ontario laboratory in relation to the *Listeria monocytogenes* outbreak, Canada, 2024 (n = 11)

Sample	Type of plant-based beverage	Beverage flavour	Best before date	Closed intact sample	Collection location	*Listeria monocytogenes* culture result	Genetically related to outbreak strain by WGS
1	Almond	Vanilla	08 Jul 24	No	Case home	Not detected	N/A
2	Coconut	Unsweetened	05 Jun 24	No	Case home	Detected	Yes
3	Coconut	Unsweetened	13 Aug 24	Yes	Retail	Not detected	N/A
4	Almond	Vanilla	28 Jul 24	No	Case home	Detected	Yes
5	Almond	Dark chocolate	29 Jul 24	No	Case home	Detected	Yes
6	Coconut	Unsweetened	27 Aug 24	Yes	Retail	Not detected	N/A
7	Coconut	Unsweetened	13 Aug 24	Yes	Retail	Not detected	N/A
8	Almond	Unsweetened	15 Aug 24	No	Case home	Detected	Yes
9	Almond	Dark chocolate	29 Jul 24	No	Case home	Not detected	N/A
10	Almond	Dark chocolate	29 Jul 24	No	Case home	Detected	Yes
11	Almond	Original	14 Sep 24	No	Case home	Detected	Yes

N/A: not applicable; WGS: whole genome sequencing.

*Listeria* was also not detected in numerous Line 1 retention samples obtained by CFIA from the Manufacturing Facility X. Further, in a targeted investigation, hundreds of environmental swabs were taken including in pre- and post-pasteurisation areas on Line 1. *Listeria monocytogenes* was detected in four non-food contact surface swabs in post-pasteurisation processing areas related to Line 1. Two of the non-food contact surface *L. monocytogenes*-positive isolates (a case packer area drain and processing line drain cover) were a genetic match to the outbreak cluster. The remaining two *L. monocytogenes*-positive swabs (a holding tank lid and processing line drain trough) were not considered genetically related to each other or the outbreak strain. Further, they were not related to any other clinical isolates in the Canadian database. The source of contamination at Manufacturing Facility X was not determined. However, the manufacturing facility did not adhere to Health Canada's policy on *L. monocytogenes* in ready-to-eat foods with respect to environmental swabbing and finished product testing [29,30]. Following the food recall warning, production never resumed on the implicated line and the manufacturer has since permanently closed Manufacturing Facility X in Ontario.

### Health Canada’s laboratory testing

Following the conclusion of the investigation, Health Canada carried out testing of 16 closed retail samples of various types of Brand A beverages (i.e. almond, coconut, and oat beverages) of various flavours (i.e. unsweetened, vanilla, and dark chocolate) for the presence of *Listeria*. All samples were produced by Manufacturing Facility X on Line 1. *Listeria* was not detected in any of the closed retail samples tested. In addition, challenge tests were completed on three coconut beverage retail samples [31]. The inoculated samples included supplemented, non-supplemented and unsweetened coconut beverage. All three samples supported the growth and survival of *L. monocytogenes* and achieved ca 2 to 3 log growth after 7 days and over 8 log growth after 68 days.

## Discussion

This report describes a listeriosis outbreak linked to pasteurised plant-based beverage products. The outbreak involved 20 confirmed cases and three deaths. The outbreak strain first emerged in August 2023. However, with few cases and no leading hypothesis, the source of illness was unknown. When the strain re-emerged almost 1 year later, routine leftover food sample collection – for what was at the time considered a sporadic listeriosis case – led to the timely identification of a novel outbreak source. Owing to its high discriminatory power, routine WGS surveillance in Ontario and Canada was able to link cases that were widely distributed both temporally and geographically. Implicating a novel food item often poses investigative challenges requiring the use of various investigative techniques [7,11]. However, as demonstrated in this and other similar investigations, appropriate food sample selection and testing can expedite source implication and public health action, preventing further illness [9,13]. In this investigation, a food recall warning was issued within 2 weeks of initial case–food WGS match.

Plant-based beverages are not a known source of *L. monocytogenes* outbreaks and, because the implicated product was pasteurised, contamination was not expected. Previous outbreaks, including an Ontario outbreak linked to chocolate milk in 2015–2016, demonstrated that a product can become contaminated post-pasteurisation and support *L. monocytogenes* growth [11,12]. This experience led PHO’s laboratory to advise the local public health inspector to include pasteurised product in the leftover samples collected from the June 2024 case’s home, despite a lower perceived risk and that the food item was not prompted for on the national standardised questionnaire.

We recognise the following limitations. Firstly, multiple genetic strains of *L. monocytogenes*, including the outbreak strain, were identified at the manufacturing facility on the implicated production line. Similar investigations have reported multiple *L. monocytogenes* isolates at production facilities [4,8,9]. Secondly, while the presence of the outbreak strain at the manufacturing facility was confirmed, the source of contamination was not identified. Pasteurisation, if temperature and duration are sufficient, is effective at killing *Listeria* and no deficiencies related to the pasteurisation process were identified. The outbreak strain was found on non-food contact surfaces in post-pasteurisation processing areas along Line 1. Contamination may have therefore occurred at any point during post-pasteurisation processing. Previous investigations have demonstrated that *Listeria* can establish itself in manufacturing facilities and form biofilms and harbourage sites, which can survive for months in areas that cannot be properly cleaned or sanitised leading to product contamination [2,8,9,11]. In the Ontario chocolate milk outbreak, the source of contamination was determined to be a post-pasteurisation pump and corrective measures were implemented to prevent recurrence [11]. As production never resumed on the implicated Line 1 and the manufacturer has since closed the facility, recurrence of this outbreak strain is unlikely. Thirdly, as the source of contamination was not determined, recommendations to prevent similar outbreaks from occurring in the future – in Ontario and elsewhere – cannot be made. It was determined the manufacturing facility did not properly adhere to Health Canada's policy on *L. monocytogenes* in ready-to-eat foods, thus it is possible that adherence to the policy could have prevented the outbreak. In a 3-year targeted retail sample survey of plant-based milk alternative products that was conducted by the CFIA from 2019 to 2022, *L. monocytogenes* was not detected in any of the samples tested leading to the conclusion that this type of product was generally safe for consumption but noted the ongoing need for good food safety practices [32].

Finally, *Listeria* was not detected in any of the closed retail or retention Brand A samples tested. Only a small number of closed samples were tested relative to the volume of product implicated in the food recall warning. As demonstrated in other *L. monocytogenes* investigations and given the distribution of outbreak cases over an 11-month period, contamination of the product was likely intermittent with some product contaminated and some not [11]. The work completed by Health Canada related to this investigation as well as studies conducted elsewhere, have demonstrated that plant-based beverage products can support the growth and proliferation of *Listeria monocytogenes* when introduced, regardless of variations in ingredient composition [16,17].

## Conclusions

Leftover food sample collection and testing by WGS was instrumental in the timely identification of the source of this outbreak – with a food recall warning issued within 2 weeks of initial case–food WGS match. As part of routine case follow-up, local public health investigators should — in consultation with public health laboratories — continue to collect left over case–food samples and submit for testing, in particular for diseases with severe outcomes such as invasive listeriosis. As there is now a new suspect to consider in *L. monocytogenes* outbreak investigations, the addition of a prompt for dairy-alternative products to standardised questionnaires used for routine case follow-up may be helpful. Finally, manufacturer adherence to applicable requirements regarding *L. monocytogenes* in ready-to-eat foods, including plant-based beverage products, continues to be critical to prevent *Listeria* contamination and subsequent illnesses and/or deaths.

## Data Availability

Public Health Ontario (PHO) cannot disclose the underlying data. Doing so would compromise individual privacy contrary to PHO’s ethical and legal obligations. Restricted access to the data may be available under conditions prescribed by the Ontario *Personal Health Information Protection Act, 2004*, the Ontario *Freedom of Information and Protection of Privacy Act*, the *Tri-Council Policy Statement: Ethical Conduct for Research Involving Humans (TCPS 2 (2018))*, and PHO privacy and ethics policies. Data are available for researchers who meet PHO’s criteria for access to confidential data. Information about PHO’s data access request process is available on-line at https://www.publichealthontario.ca/en/data-and-analysis/using-data/data-requests. The whole genome sequencing datasets generated during and/or analysed during the current study are available in the National Centre for Biotechnology Information repository within BioProject PRJNA563085, https://www.ncbi.nlm.nih.gov.

## Supplementary Data

201000 Supplementary Material
