# Supplementary material for: A new suspect: Listeria monocytogenes outbreak linked to pasteurised plant-based beverages, Canada, 2024
Source: Euro Surveill. 2026 Jun 18;31(24):2500883. doi: 10.2807/1560-7917.ES.2026.31.24.2500883 (PMC13308610; doi:10.2807/1560-7917.ES.2026.31.24.2500883)
Supplement: Supplementary Material [file 25-00883_HOBBS_Supplement.pdf]

Public Health Ontario | Santé publique Ontario

# Supplement S1

This supplementary material is hosted by *Eurosurveillance* as supporting information alongside the article 'A new suspect: *Listeria monocytogenes* outbreak linked to pasteurised plant-based beverages, Canada, 2024', on behalf of the authors, who remain responsible for the accuracy and appropriateness of the content. The same standards for ethics, copyright, attributions and permissions as for the article apply. Supplements are not edited by *Eurosurveillance* and the journal is not responsible for the maintenance of any links or email addresses provided therein.

## *Listeria monocytogenes*: Plant-based dairy alternative products focused questionnaire

| |
|---|
| **Please complete this questionnaire for *L. monocytogenes* cases with the outbreak whole genome sequencing (WGS) strain or where WGS is pending.** |
| **Case ID:** |

| **Section 1: Interviewer details** | |
|---|---|
| **Interviewed by:** | **Date of interview:** d ____ /m _____ /2024 |
| **Respondent was:** ☐case ☐parent ☐spouse ☐caretaker ☐other, specify: | |
| **Date of illness onset date:** d ____ /m _____ /y_____ | |

| **Section 2: Special diets** |
|---|
| **Do you have any special diets (e.g. vegan, vegetarian)?**<br>☐yes ☐no ☐don't know ☐not asked<br>**Notes:** |
| **Do you have any dietary restrictions/allergies (e.g. lactose intolerance):**<br>☐yes ☐no ☐don't know ☐not asked<br>**Notes:** |

| Section 3: Food history |
|---|
| **Instructions for interviewer:**<br><br>For each food item that the case consumed, please ask follow up questions regarding the brand, location of purchase, etc. Please read all response options to the case. |
| **Instructions for case:**<br><br>I am interested in any dairy-free or plant-based dairy-alternative products you may have consumed in the 4 weeks before your illness onset date, that is, from d____/m____/y____ through d____/m____/y____ .<br><br>For each food item, please give me your best guess as to whether you ATE the food, you're not sure but you PROBABLY ate the food, or you DID NOT EAT the food.<br><br>*Cases have occurred up to 70 days following exposure to a contaminated product. |
| **Did you drink any dairy-free or plant-based dairy alternative products?**<br><br>☐yes ☐no ☐probably ☐don't know ☐not asked |

| | |
|---|---|
| **Oat-based dairy alternative products?**<br><br>☐yes ☐no ☐probably ☐don't know ☐not asked | **Brand:**<br><br>**Products details (flavour, package size, was it sold at room temperature or refrigerated?):**<br><br>**Best before date:**<br><br>**Purchase location (store name and intersection):**<br><br>**Does the store have a loyalty card program?**<br>☐yes ☐no ☐don't know<br><br>**If yes, do you consent to loyalty card data collection?**<br>☐yes ☐no ☐don't know<br><br>**Date of purchase:**<br><br>**Are you able to provide photos of product packaging (all sides)?**<br>☐yes ☐no ☐don't know |
| **Rice-based dairy alternative products?**<br><br>☐yes ☐no ☐probably ☐don't know ☐not asked | **Brand:**<br><br>**Products details (flavour, package size, was it sold at room temperature or refrigerated?):**<br><br>**Best before date:**<br><br>**Purchase location (store name and intersection):**<br><br>**Does the store have a loyalty card program?**<br>☐yes ☐no ☐don't know<br><br>**If yes, do you consent to loyalty card data collection?**<br>☐yes ☐no ☐don't know<br><br>**Date of purchase:**<br><br>**Are you able to provide photos of product packaging (all sides)?**<br>☐yes ☐no ☐don't know |

| | |
|---|---|
| **Soy-based dairy alternative products?**<br><br>☐yes ☐no ☐probably ☐don't know ☐not asked | **Brand:**<br><br>**Products details (flavour, package size, was it sold at room temperature or refrigerated?):**<br><br>**Best before date:**<br><br>**Purchase location (store name and intersection):**<br><br>**Does the store have a loyalty card program?**<br>☐yes ☐no ☐don't know<br><br>**If yes, do you consent to loyalty card data collection?**<br>☐yes ☐no ☐don't know<br><br>**Date of purchase:**<br><br>**Are you able to provide photos of product packaging (all sides)?**<br>☐yes ☐no ☐don't know |
| **Coconut-based dairy alternative products?**<br><br>☐yes ☐no ☐probably ☐don't know ☐not asked | **Brand:**<br><br>**Products details (flavour, package size, was it sold at room temperature or refrigerated?):**<br><br>**Best before date:**<br><br>**Purchase location (store name and intersection):**<br><br>**Does the store have a loyalty card program?**<br>☐yes ☐no ☐don't know<br><br>**If yes, do you consent to loyalty card data collection?**<br>☐yes ☐no ☐don't know<br><br>**Date of purchase:**<br><br>**Are you able to provide photos of product packaging (all sides)?**<br>☐yes ☐no ☐don't know |

| | |
|---|---|
| **Nut-based dairy alternative products (e.g. cashew/almond-based products)?**<br><br>☐yes ☐no ☐probably ☐don't know ☐not asked | **Brand:**<br><br>**Products details (flavour, package size, was it sold at room temperature or refrigerated?):**<br><br>**Best before date:**<br><br>**Purchase location (store name and intersection):**<br><br>**Does the store have a loyalty card program?**<br>☐yes ☐no ☐don't know<br><br>**If yes, do you consent to loyalty card data collection?**<br>☐yes ☐no ☐don't know<br><br>**Date of purchase:**<br><br>**Are you able to provide photos of product packaging (all sides)?**<br>☐yes ☐no ☐don't know |
| **Other dairy-alternative products?**<br><br>☐yes ☐no ☐probably ☐don't know ☐not asked | **Brand:**<br><br>**Products details (flavour, package size, was it sold at room temperature or refrigerated?):**<br><br>**Best before date:**<br><br>**Purchase location (store name and intersection):**<br><br>**Does the store have a loyalty card program?**<br>☐yes ☐no ☐don't know<br><br>**If yes, do you consent to loyalty card data collection?**<br>☐yes ☐no ☐don't know<br><br>**Date of purchase:**<br><br>**Are you able to provide photos of product packaging (all sides)?**<br>☐yes ☐no ☐don't know |
